# Autoantibodies against mono- and tri-methylated lysine display similar but also distinctive characteristics

**DOI:** 10.1371/journal.pone.0172166

**Published:** 2017-02-21

**Authors:** Zhiqiang Wang, Younan Ma, Fan Liu, Linjie Chen, Ruitong Gao, Wei Zhang

**Affiliations:** 1 Department of Immunology, Institute of Basic Medical Sciences, Chinese Academy of Medical Sciences, School of Basic Medicine, Peking Union Medical College, Beijing China; 2 Biomolecular Mass Spectrometry and Proteomics, Bijvoet Centre for Biomolecular Research and Utrecht Institute for Pharmaceutical Sciences, University of Utrecht, Utrecht, The Netherlands; 3 Netherlands Proteomics Center, Utrecht, The Netherlands; 4 Department of Rheumatology and Immunoloy, the First Affiliated Hospital of Bengbu Medical College, Bengbu, China; 5 Department of Nephrology, Peking Union Medical College Hospital, Chinese Academy of Medical Sciences, Peking Union Medical College, Beijing, China; Duke University School of Medicine, UNITED STATES

## Abstract

Autoantibodies can be either harmful or beneficial to the body. The beneficial autoantibodies play important roles in immunosurveillance, clearance of body waste and maintenance of immune homeostasis. Despite their importance, however, people’s knowledge on the protective autoantibodies is still very limited. In the current study, we examined two autoantibodies that recognized epitopes with only one amino acid. One was against mono-methylated lysine (Kme) and the other was against tri-methylated lysine (Kme3). We found that the antibodies were highly specific and not polyreactive. They did not cross-react each other. Although anti-Kme antibodies were IgM only, a large proportion of the anti-Kme3 antibodies were switched to the IgG isotype. Mass spectrometric analysis showed that both of the antibodies were mainly derived from IGHV 3–7 and/or IGHV3-74 germ line genes with conserved CDR2. *De novo* sequencing showed that there was a mutation at either of the SS positions on the CDR1 region, which changed one of the serine residues to a basic amino acid, i.e., arginine or lysine. We also found that neither of the antibodies was expressed at birth, and their earliest appearance was approximately 5 months after birth. All healthy human beings expressed the antibodies when they reached age two and maintained the expression thereafter throughout their life. Patients with systemic lupus erythematosus had lower levels of the IgM isotype antibodies. Serum levels of the two IgM antibodies were closely correlated, implying that they were produced by cells from the same B cell subset. We also found that both anti-Kme and anti-Kme3 antibodies could bind and might take part in the clearance of neutrophil extracellular traps released from activated cells. In conclusion, although anti-Kme and anti-Kme3 antibodies share many similarities in their origins, they are different antibodies and have different characteristics.

## Introduction

Autoantibodies are antibodies that react to body self components. They can be divided into natural antibodies and immune antibodies. Natural autoantibodies (NAAs) are produced without exogenous antigen stimulation [1]. They can be detected in cord blood and in mice housed under germ-free conditions and fed an antigen-free diet [2]. NAAs are usually of the IgM isotype and are polyreactive, which means that they can bind several unrelated antigens with moderate affinity [3]. Immune autoantibodies are produced in response to foreign antigens and become self-reactive by mechanisms such as molecular mimicry and epitope spreading [4]. B cells in primary immune responses produce antibodies mainly of the IgM isotype, and they switch to IgG and other antibody isotypes during secondary and subsequent immune responses.

Although autoantibodies can react to body self components and may cause severe consequences and even threaten life under pathological conditions [5], they are not always harmful and can be even helpful for the body. Most known beneficial autoantibodies are NAAs [6]. They participate in the clearance of aging cells, cellular debris, altered self on cells and plasma components [7]. They also play an important role in anti-tumor surveillance and in the selection of immune repertoires and maintenance of immune homeostasis [8]. It has been found that there is a negative correlation between anti-dsDNA IgM antibodies and glomerulonephritis [9]. Systemic lupus erythematosus (SLE) patients with low disease activity tended to have higher levels of polyreactive IgM antibodies [10]. It was reported that expression of IgM NAAs in MRL-lpr mice prevented proteinuria and reduced kidney immune complexes. These mice showed a significant reduction in glomerulonephritis and a dramatic increase in survival [2,11]. Thus, it can be predicted that protective autoantibodies may have therapeutic potential. However, in spite of their importance, our knowledge of the nature of protective autoantibodies is still very limited.

In previous work, we identified IgM autoantibodies that reacted to a very small epitope with only one amino acid, i.e., mono-methylated lysine (Kme) [12]. The antibodies were not polyreactive. They recognized Kme on any peptides without sequence preferences, and the binding could be completely inhibited by ε-amine mono-methylated lysine. The antibodies were present in healthy subjects, and their levels in patients with SLE were significantly lower.

In the present work, we further studied the nature of anti-Kme antibody. In addition, we identified another antibody that recognized tri-methylated lysine (Kme3). Comparison of the two antibodies revealed that they share many similarities in their origin and function. Nevertheless, the two antibodies possess different antigen specificities and isotype priorities.

## Materials and methods

### Ethics statement

This study was performed in accordance with the Declaration of Helsinki and approved by the ethic committees of Institute of Basic Medical Sciences, Chinese Academy of Medical Sciences (Approval ID 012–2012, Institutional Review Board of IBMS, CAMS). All the personal privacy was well protected throughout the work. All the serum samples used in this work were leftover samples after clinical examinations of patients or routine health check of healthy people. As these samples were treated as abandoned samples and used in anonymous codes, the informed consent was exempted. Patient’s clinical information was obtained through doctors who had the master keys of coding and would strictly abide by the confidentiality agreement. Sera used for antibody purification were from volunteers of our laboratory.

### Antibodies and peptides

Monoclonal antibodies to human IgM (KT16, KT38), IgG (KT47, KT48), IgA (KT41), IgE (1A2), J chain (KT109) and lactoferrin (KT14) were supplied by Absea Biotechnology Ltd (Beijing, China). HRP conjugated goat anti-mouse IgG polyclonal antibody (A2554) were purchased from Sigma-Aldrich (St. Louis, MO, USA). FITC-labeled goat anti-mouse IgG polyclonal antibody was purchased from ZSGB-BIO (Beijing, China). Anti-NP IgG and IgM monoclonalantibodies were purified from culture supernatants. Peptides (Table 1) were synthesized by Scilight-peptide Inc (Beijing, China) and conjugated to bovine serum albumin (BSA) by Absea. GGK, GGKme and GGKme3 peptides coupled to Sepharose beads were prepared by Absea.

**Table 1:** Peptides and their modifications.

Abbreviations	Sequences and modifications
H3_1-19_	ARTKQTARKSTGGKAPRKQC
H3_41-55_	YRPGTVALREIRRYQC
H3_1-19_K4me	ARTK(me)QTARKSTGGKAPRKQC
H3_1-19_K4me2	ARTK(me2)QTARKSTGGKAPRKQC
H3_1-19_K4me3	ARTK(me3)QTARKSTGGKAPRKQC
H3_1-19_K9me	ARTKQTARK(me)STGGKAPRKQC
H3_1-19_K9me2	ARTKQTARK(me2)STGGKAPRKQC
H3_1-19_K9me3	ARTKQTARK(me3)STGGKAPRKQC
H3_22-36_K27me3	TKAARK(me3)SAPATGGVKC
GGK	GGKGGSGGSGGSGC
GGKme	GGK(me)GGSGGSGGSGC
GGKme3	GGK(me3)GGSGGSGGSGC
H3_1-19_K9ac	ARTKQTARK(ac)STGGKAPRKQC
H3_22-36_S28ph	TKAARKS(ph)APATGGVKC
H3_1-19_R8me	ARTKQTAR(me)KSTGGKAPRKQC

### Serum samples

Sera for antibody purification were from healthy volunteers in the laboratory. Sera from healthy subjects were from people who undergone routine health checkups. Sera from babies aged 1 month to 2 years old were from the Outpatient Department at the Maternity Hospital of San He, Hebei Province, China. None of these subjects had any rheumatologic conditions when recruited.

Sera from patients with rheumatic diseases were collected from the Department of Rheumatology and Immunology, the First Affiliated Hospital of Bengbu Medical College, Anhui Province, China, and from the Department of Nephrology, Peking Union Medical College Hospital, Chinese Academy of Medical Sciences, Peking Union Medical College, Beijing, China. They were 42 SLE patients, 23 rheumatoid arthritis (RA) patients, 16 Ankylosing spondylitis patients and 6 Sjogren's syndrome patients. All of the SLE patients met at least 4 classification criteria from the American College of Rheumatology [13]. The patients information is listed in Table 2.

**Table 2:** Patients' information and treatments.

Disease	Number	Gender (Male/ Female)	AverageAge (Year)	Age Range (Year)	Medication			
					GC (+/-)	IS (+/-)	AM (+/-)	IVIG (+/-)
SLE	42	3/39	33.5±11.4	19–67	39/3	28/14	23/19	6/36
RA	23	2/21	46.9±13.8	26–75	12/11	22/1	12/11	0/23
AS	16	15/1	33.3±13.5	18–64	7/9	12/4	0/16	0/16
SS	6	1/5	49±10.6	30–62	5/1	6/0	6/0	0/6

GC, Glucocorticoids, such as prednisone, dexamethason, hydrocortisone; IS, Immunosuppressants, such as cyclophosphamide, cyclosporin A, methotrexate; AM,Antimalarials, such as chloroquine, hydroxychloroquine; IVIG, Intravenous immunoglobin.

All sera were collected from clotted blood and stored at -80°C until use.

This study was approved by the ethic committee of Institute of Basic Medical Sciences, Chinese Academy of Medical Sciences (Approved ID 012–2012, Institutional Review Board of IBMS, CAMS).

### Tandem affinity purification of anti-Kme and anti-Kme3 antibodies

Serum (15 mL) was first absorbed with 0.8 mL Sepharose beads coupled with GGK peptide at 4°C overnight with rotation. After separating the beads with the serum using microcentrifuge spin columns (Pierce, Rockford, IL, USA), the passing through serum was first absorbed with 0.8 mL beads coupled with GGKme peptide and then with beads coupled with GGKme3 peptide. After washed with PBS and 1 M NaCl, the beads were separately eluted with 0.1 M glycine, pH 2.5, and the eluates were neutralized immediately.

### ELISA

Ninety-six-well microtiter plates (Millipore, Billerica, MA, USA)were coated with peptides cross-linked to BSA (1 μg/mL according to the BSA concentration, 100 μL/well) at 4°C overnight in coating buffer containing 15 mM Na_2_CO_3_, 35 mM NaHCO_3_, pH 9.6. After washed with PBS containing 0.05% Tween-20 (PBST), the plates were blocked with 2% BSA. Then, 100 μL/well of purified antibodies (1.4 μg/mL) or serum samples (1:100 diluted) were added and incubated at RT for 1 h. After washing, the mouse anti-human IgG monoclonal antibody (KT47, 1:1000 diluted) or mouse anti-human IgM monoclonal antibody (KT16, 1:1000 diluted) was added and incubated at RT for 1 h. Then, the plates were washed, and HRP-conjugated goat anti-mouse IgG (1:5000 diluted)was added. After incubating at RT for 1 h, the wells were washed, and the substrate ABTS (Amresco, Solon, OH, USA) was added. After color developed, OD values were read at 405 nm.

The polyreactivity tests were performed as described by Tiller et al[14].Briefly 100 μL/well of dsDNA, ssDNA and LPS (Sigma-Aldrich) at 10 μg/mL and insulin (Wako, Osaka, Japan) at 5 μg/mL were coated. After washing and blocking, the plates were incubated with affinity purified antibodies first and then with KT47 anti-human IgG(1:1000 diluted) or with KT16anti-human IgM (1:1000 diluted). HRP conjugated goat anti-mouse IgG was used to detect bound antibodies.

Serum IgM and IgG concentrations were measured by sandwich ELISA. To measure IgM, KT16 (5 μg/mL)was used as the capture antibody and HRP-conjugated KT38 (1:1000 diluted) was used as the detection antibody. To measure IgG, KT48 (5 μg/mL)was used as the capture antibody and HRP-conjugated KT47(1:1000 diluted) was used as the detection antibody.

### Mass spectrometric (MS) analysis for anti-Kme and anti-Kme3 heavy chains

The tandem affinity purified antibodies were separated by SDS-PAGE under reducing conditions. After stained by Coomassie blue, the bands corresponding to heavy chains of IgG and IgM were excised from the gel. The proteins in the gel plugs were reduced by dithiothreitol and alkylated with iodoacetamide. Subsequent in-gel trypsin digestion was performed at 37°C overnight, and the digested peptides were extracted and dried. The samples were analyzed by the TripleTOF™ 5600LC/MS/MS high resolution mass spectrometric system (AB SCIEX™, Framingham, MA, USA) at the Central Laboratory, Institute of Basic Medical Sciences, Chinese Academy of Medical Sciences.The peptide sequences were identified using ProteinPilot (version 4.5, AB SCIEX™, Framingham, MA, USA). All identified Ig peptide sequences were aligned to human Ig germ line sequences derived from the International ImMunoGeneTics Information System (IMGT) database (Montpellier, France). The following settings were used in the search: precursor and product ion mass tolerance: 0.05 Da; fixed modification: Cys carbamidomethyl; variable modification: Met oxidation. Peptide results were filtered using an average score cutoff of 99% via ProteinPilot.

LC/MS experiments for *de novo* sequencing were performed on an Orbitrap Fusion (Thermo Fisher Scientific, Bremen, Germany) mass spectrometer with both MS and HCD MS2 acquisitions acquired in the Orbitrap. *De novo* sequencing was performed using PEAKS studio 7.0 (Bioinformatics Solutions Inc, ON, Canada). The following settings were used in the search: precursor ion mass tolerance: 10 ppm; product ion mass tolerance: 0.02 Da; fixed modification: Cys carbamidomethyl; variable modification: Met oxidation.*De novo* sequencing results were filtered using an average local confidence (ALC) score cutoff of 80%.

### Neutrophil extracellular traps (NETs) induction and immunofluorescence staining

Neutrophils were isolated from heparinized blood of healthy donors by sedimentation using Dextran T500 and density centrifugation through discontinuous Percoll gradients (GE Healthcare Bio-Sciences AB, Uppsala, Sweden). Neutrophils (2×10^5^/well) were seeded on poly-L-lysine-coated cover slips in 24-well plates in present of 25 nM of PMA (Sigma) and incubated for 4 h at 37°C in a CO_2_ incubator. The slides were then fixed with 4% paraformaldehyde and blocked with PBS containing 2% BSA at RT for 30 min. The fixed cells were incubated with KT14 anti-lactoferrin antibody (1:1000 diluted), anti-Kme or anti-Kme3 antibody (1 μg/mL) at RT for 1 h. Anti-NP IgG (1 μg/mL) and anti-NP IgM (1 μg/mL) were used as negative controls. The bound IgG or IgM was detected using KT16 (1:1000 diluted) or KT47 (1:1000 diluted) followed by FITC-labeled anti-mouse antibody (1:200 diluted). Cells were observed in PBS containing PI (Sigma) using an Olympus IX71 fluorescence microscope (Olympus, Tokyo, Japan).

### Statistics

All data were analyzed using GraphPad Prism (Version 5.01, La Jolla, CA, USA). A two-tailed unpaired *t* test was performed to analyze the differences between two groups. Pearson test was performed to analyze correlations between data with a normal distribution, and Spearman analysis was performed to analyze correlations between data with non-normal distribution. Significance was noted as *P< 0.05; **P< 0.01; ***P< 0.001.

## Results

### Antibodies against Kme and Kme3 are highly specific

To characterize antibodies against Kme and Kme3, we performed affinity purification using tandem columns. Sera (15 mL) from healthy subjects were first absorbed with an artificially designed peptide GGK with the sequence GGKGGSGGSGGSGC and then with lysine mono-methylated GGK (GGKme) or tri-methylated GGK (GGKme3). Antibodies bound to the columns were separately eluted. There were essentially no antibodies eluted from the GGK column, and the amounts of antibodies obtained from the GGKme and GGKme3 columns on average were 75 μg and 110 μg, respectively.

**Fig 1:**
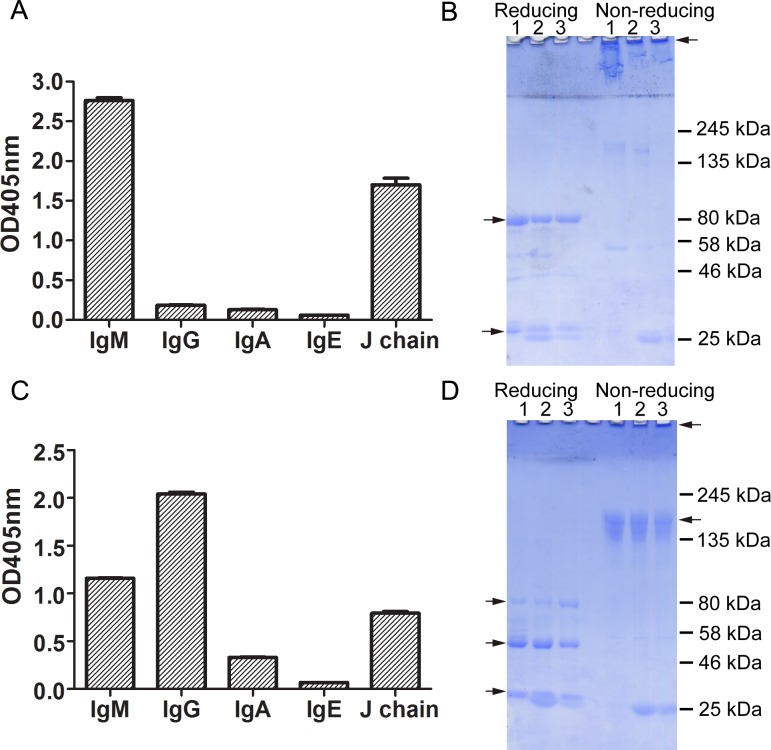
Isotypes and molecular masses of anti-Kme and anti-Kme3 antibodies. Anti-Kme and anti-Kme3 antibodies were affinity purified using tandem columns. Antibodies bound to GGKme and GGKme3 beads were separately eluted (see Materials and Methods) and tested by ELISA for their isotypes and J chain protein. Mouse monoclonal antibodies against human IgM (KT16), IgG (KT47), IgA (KT41), IgE (1A2), J chain (KT109) were used as primary antibodies. HRP conjugated goat anti-mouse IgG (Fc specific) was used as a secondary antibody. (A) Isotypes and J chain of antibodies eluted from the GGKme column. (B) SDS-PAGE of antibodies eluted from the GGKme column under reducing and non-reducing conditions. (C) Isotypes and J chain of antibodies eluted from the GGKme3 column. (D) SDS-PAGE of antibodies eluted from the GGKme3 column under reducing and non-reducing conditions. Arrows indicate molecular mass of IgG and IgM under non-reducing conditions and heavy and light chains of IgG and IgM under reducing conditions.

The antibodies were then characterized by ELISA and SDS-PAGE. Fig 1 shows the isotypes and molecular masses of antibodies from three randomly selected healthy subjects. Almost all antibodies eluted from the GGKme column were of the IgM isotype that could be detected by both anti-IgM and anti-J-chain antibodies (Fig 1A). The IgM could not enter the resolving gel under non-reducing conditions and showed a heavy chain band around 80 kDa and a light chain band around 27 kDa under reducing conditions (Fig 1B). In comparison, antibodies eluted from the GGKme3 column had not only IgM but also a large proportion of IgG and a small proportion of IgA (Fig 1C). J chain associated with polymeric IgA and IgM could also be detected. The SDS-PAGE results showed two heavy chain bands with molecular masses around 50 kDa and 80 kDa representing IgG and IgM, respectively, under reducing conditions (Fig 1D).

**Fig 2:**
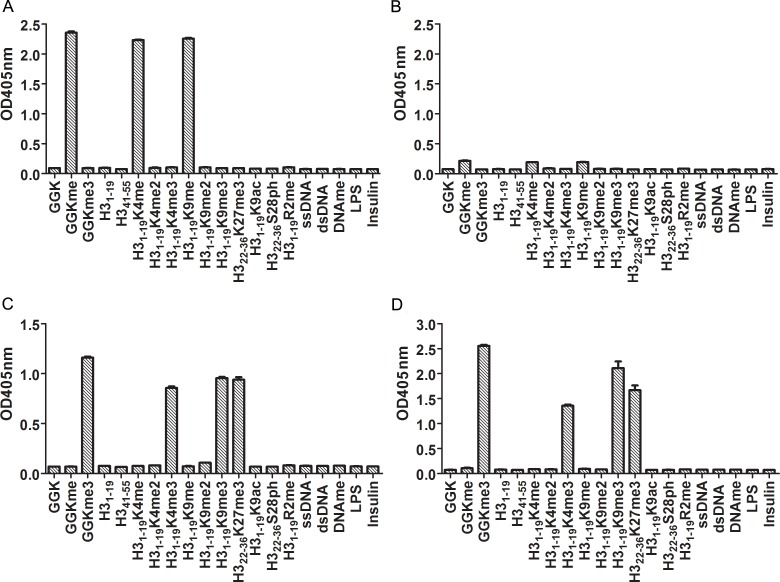
Specificities of anti-Kme and anti-Kme3 antibodies. Ninety-six-well plates were coated with various antigens as indicated. Affinity purified anti-Kme and anti-Kme3 antibodies were tested by ELISA for their reactivity to the coated antigens (see Materials and Methods). Anti-Kme and anti-Kme3 antibodies were detected using KT47 anti-human IgG and KT16 anti-human IgM as primary antibodies and HRP conjugated goat anti-mouse IgG (Fc specific) as a secondary antibody. (A) Antibodies were eluted from the GGKme column and detected by KT16 anti-human IgM antibody. (B) Antibodies were eluted from the GGKme column and detected by KT47 anti-human IgG antibody. (C) Antibodies were eluted from the GGKme3 column and detected by KT16 anti-human IgM antibody. (D) Antibodies were eluted from the GGKme3 column and detected by KT47 anti-human IgG antibody.

The purified antibodies were highly specific. We previously showed that IgM anti-Kme reacted to peptides that contained mono-methylated lysine and did not react to peptides that had no methylation or had di- or tri-methylated lysine [12]. To determine whether the IgM reacted with methyl groups on other residues, we tested its reactivity to methylated DNA and mono-methylated arginine that was on a peptide corresponding to the 1–19 N-terminal residues of histone H3 (H3_1-19_R2me, Table 1). The results showed that the IgM did not react to any of them, indicating that the IgM was indeed specifically against Kme (Fig 2A). Thus, mono-methylated lysine was the sole epitope of IgM anti-Kme. The antibodies eluted from the GGKme column had very little IgG against peptides containing Kme (Fig 2B).

We also tested specificities of anti-Kme3 using a set of peptides, i.e., GGK peptide with non-, mono- and tri-methylation, histone 3 N-terminal peptides without modification or modified with phosphorylation, acetylation and various degrees of methylations (Table 1). Our results showed that both IgG and IgM anti-Kme3 recognized peptides containing Kme3 and did not recognize any other peptides that had the same amino acid sequences but different modifications (Fig 2C and 2D). Thus tri-methylated lysine was the sole epitope of IgG and IgM anti-Kme3.

We also tested the polyreactivity of anti-Kme and anti-Kme3 antibodies, and none of them reacted with ssDNA, dsDNA, LPS or insulin (Fig 2A–2D).

### MS analysis of heavy chains of anti-Kme and anti-Kme3 antibodies

To investigate the origin of anti-Kme and anti-Kme3 antibodies, we performed an MS analysis for the use of germ line V gene segments of the heavy chains. Affinity purified anti-Kme and anti-Kme3 antibodies from three healthy subjects were analyzed.

The number of matched peptides found from the IMGT databasefor variable and constant regions of heavy chains in different families is shown in Table 3. The variable regions of IgM anti-Kme antibodies were mainly from the IGHV3 gene family, and some of the peptides were also derived from the IGHV2 gene family. For anti-Kme3 antibodies, both variable regions of IgM and IgG were predominantly from the IGHV3 gene family. Peptides belonging to other families were trivial and could be neglected. IgG1, IgG2 and IgG3 but not IgG4 subclasses were identified (Table 3).

**Table 3:** The number of matched peptides in IGHV and IGHC subgroups.

Location		IgM anti-Kme			IgM anti-Kme3			IgG anti-Kme3		
		1#	2#	3#	1#	2#	3#	1#	2#	3#
IGHV	Family 1	22	30	19	5	13	16	10	13	12
	Family 2	9	43	121	0	0	0	0	0	0
	Family 3	58	88	169	338	245	272	140	161	285
	Family 4	18	25	13	18	26	44	56	11	27
	Family 5	8	6	12	0	0	0	0	0	0
	Family 6	3	4	4	0	2	0	0	0	0
	Family 7	2	5	5	0	0	4	0	0	0
IGHC	IGHM	99	152	259	82	118	114	5	10	3
	IGHA1	1	5	3	3	4	0	0	3	3
	IGHA2	0	0	0	2	0	0	0	3	0
	IGHG1	1	0	0	5	4	16	60	43	53
	IGHG2	1	5	4	5	5	17	87	56	68
	IGHG3	1	4	0	5	5	16	54	0	45
	IGHG4	1	0	0	0	0	0	0	0	0
Total		224	367	609	463	422	499	412	300	496

IGHV, immunoglobulin heavy chainvariable region; IGHC, immunoglobulin heavy chain constant region.

**Fig 3:**
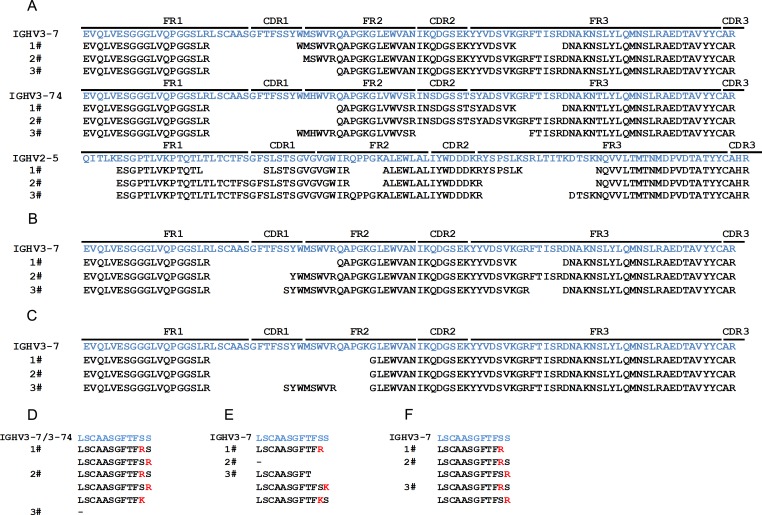
Alignment of IGHV genes with peptides from the variable regions of anti-Kme and anti-Kme3 antibodies. The heavy chains of affinity purified IgM anti-Kme and IgG and IgM anti-Kme3 from three healthy subjects were analyzed by MS. The peptide sequences responding to variable regions of immunoglobulin heavy chains were identified using ProteinPilot and aligned with IGHV sequences from the IMGT database. To search the CDR1 of the antibodies, *de novo* sequencing was performed using heavy chains of IgM anti-Kme and anti-Kme3 purified from a fourth donor. The obtained CDR1 sequences were used as a template to search MS data from the first three donors. (A) Identified peptides from the variable regions of IgM anti-Kme antibodies. (B) Identified peptides from the variable regions of IgM anti-Kme3 antibodies. (C) Identified peptides from the variable regions of IgG anti-Kme3 antibodies. (D) CDR1 regions of IgM anti-Kme antibodies. (E) CDR1 regions of IgM anti-Kme3 antibodies. (F) CDR1 regions of IgG anti-Kme3 antibodies. The corresponding germ line sequences are shown in blue, and the CDR1 mutations are shown in red.

The sequence coverage of the identified IgM anti-Kme heavy chain variable region to the germ line sequences is shown in Table 4. The percentages of total coverage on the IGHV3-7, IGHV3-74 and IGHV2-5 germ line genes were 85.7%, 85.7% and 88% respectively (Fig 3A, Table 4). Peptides from other gene segments were also identified, but their coverage was much smaller than that of the IGHV3-7, IGHV3-74 and IGHV2-5 genes (Table 4). The MS did not identify peptides that were located within the CDR1 and adjacent FR1 region, and only two amino acids (AR) were identified in the CDR3 region (Fig 3A). For variable regions derived from IGHV2-5, the major parts that were not identified were located in the FR1, FR2 and FR3 regions.

**Table 4:** Sequence coverage of anti-Kme and anti-Kme3 antibodies in IGHV subgroups.

	Gene segment	Number of amino acids in the region	1#		2#		3#	
			Number of identified amino acids	Sequence coverage	Number of identified amino acids	Sequence coverage	Number of identified amino acids	Sequence coverage
IgM anti-Kme antibodies	IGHV3-7	98	78	79.6%	84	85.7%	79	80.6%
	IGHV3-74	98	72	73.5%	79	80.6%	68	69.4%
	IGHV2-5	100	70	70.0%	74	74.0%	82	82.0%
	IGHV3-23	98	41	41.8%	51	52.0%	49	50.0%
	IGHV3-53	97	41	42.3%	45	46.4%	49	50.5%
	IGHV3-66	97	41	42.3%	45	46.4%	49	50.5%
	IGHV3-35	98	30	30.6%	30	30.6%	38	38.8%
IgM anti-Kme3 antibodies	IGHV3-7	98	72	73.5%	86	87.8%	81	82.7%
	IGHV3-15	100	61	61.0%	46	46.0%	53	53.0%
	IGHV3-33	98	54	55.1%	68	69.4%	48	49.0%
	IGHV3-23	98	45	45.9%	61	62.2%	60	61.2%
	IGHV3-53	97	45	46.4%	73	75.3%	41	42.3%
	IGHV3-30	98	54	55.1%	68	69.4%	48	49.0%
	IGHV3-9	99	45	45.5%	53	53.5%	41	41.4%
IgG anti-Kme3 antibodies	IGHV3-7	98	73	74.5%	73	74.5%	81	82.7%
	IGHV3-74	98	52	53.1%	48	48.9%	52	53.1%
	IGHV3-43	99	56	65.6%	39	39.4%	48	48.5%
	IGHV3-30	98	60	61.2%	48	49.0%	64	65.3%
	IGHV3-35	98	55	56.1%	41	41.8%	30	30.6%
	IGHV3-15	100	47	47.0%	51	51.0%	59	59.0%
	IGHV3-33	98	60	61.2%	48	49.0%	64	65.3%

The situations for the IgM anti-Kme3 antibodies were clearer. The sequence coverage of the IgM and IgG anti-Kme3 heavy chain variable region to the germ line sequences is shown in Table 4. The variable regions of the IgG and the IgM were essentially the same. Their coverage on IGHV3-7 was 87.8% for IgM anti-Kme3 and 82.7% for IgG anti-Kme3. The identified peptides covered the full germ line CDR2 but did not cover CDR1, adjacent FR1 or CDR3 (Fig 3B and 3C).

The germ line sequence of IGHV3-7/74 corresponding to the missing CDR1 and adjacent FR1 was LSCAASGFTFSSYW. To further search the CDR1 sequences of the antibodies, we performed *de novo* sequencing using IgM anti-Kme and anti-Kme3 purified from a fourth donor. Several peptides were identified by *de novo* sequencing, and each had mutations compared with the germ line sequence (Table 5). We then used the obtained sequences as templates to search the MS data of the original three donors and found that IgM anti-Kme and both IgG and IgM anti-Kme3 antibodies had a single mutation at position SS, resulting in a substitution of a basic amino acid (arginine or lysine) to either of the double serine residues (Fig 3D–3F).

**Table 5:** Identified mutations corresponding to sequence LSCAASGFTFSSYW.

Peptides	Length	ALC (%)	m/z	z	Mass	ppm	PTM	local confidence (%)
LSCAASGFTFK	11	93	594.79	2	1187.565	0.9	Carbamidomethylation	89 95 99 97 94 97 80 90 97 99 95
LSCAASGFTFR	11	93	608.7932	2	1215.571	1.1	Carbamidomethylation	93 96 99 99 99 94 85 92 92 95 89
LSCAASGFTFSR	12	93	652.309	2	1302.603	0.7	Carbamidomethylation	92 96 99 99 99 95 85 92 92 90 92 91
LSCAASGFSFK	11	91	587.7822	2	1173.549	0.8	Carbamidomethylation	88 94 99 96 95 96 76 88 89 93 93
LSCAASGFTFSK	12	88	638.306	2	1274.596	0.7	Carbamidomethylation	85 93 99 96 94 90 77 88 94 95 85 57
LSCVASFGR	9	88	498.7504	2	995.4858	0.4	Carbamidomethylation	92 95 99 95 93 89 85 82 60
LSCAAYQR	8	84	484.736	2	967.4545	2.9	Carbamidomethylation	91 94 95 92 83 67 79 74

### Serum levels of IgM anti-Kme and anti-Kme3 are correlated

**Fig 4:**
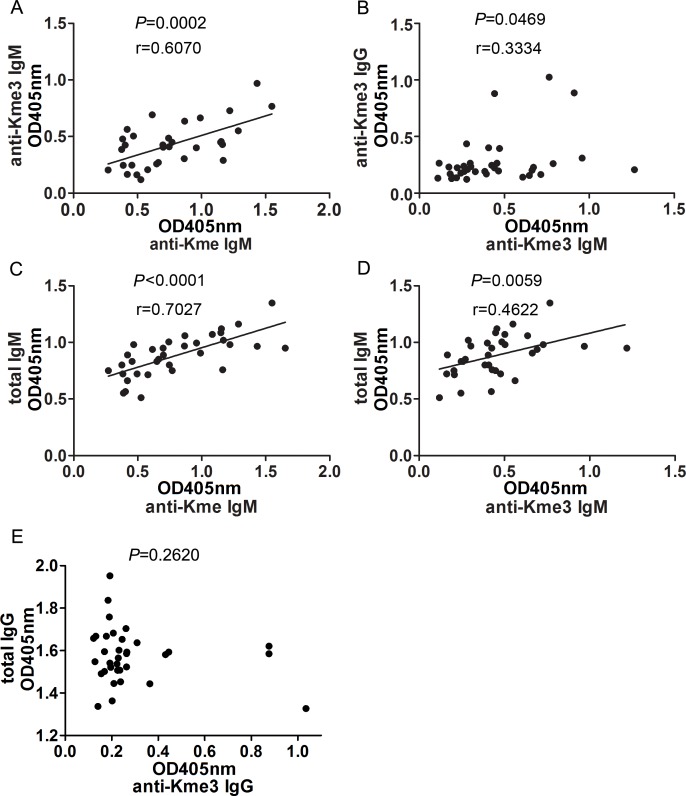
Correlations between anti-Kme and anti-Kme3 antibodies with each other and with total IgG or IgM. Serum levels (n = 34) of IgM and IgG anti-Kme and anti-Kme3 antibodies were measured by ELISA using GGKme and GGKme3 conjugated BSA to coat 96-well plates. Anti-Kme and anti-Kme3 antibodies were detected using KT47 anti-human IgG and KT16 anti-human IgM as primary antibodies and HRP conjugated goat anti-mouse IgG as a secondary antibody. Serum levels of total IgG and IgM were measured by sandwich ELISA using KT48 and HRP conjugated KT47 for IgG and KT16 and HRP conjugated KT38 for IgM (See Materials and Methods). The correlations were analyzed by Pearson correlation analysis. (A) Correlations between IgM anti-Kme and IgM anti-Kme3 in serum. (B) Correlations between IgG anti-Kme3 and IgM anti-Kme3 in serum. (C) Correlations between IgM anti-Kme and total IgM in serum. (D) Correlations between IgM anti-Kme3 and total IgM in serum. (E) Correlations between IgG anti-Kme3 and total IgG in serum.

Although anti-Kme and anti-Kme3 antibodies recognized different epitopes and there was no cross-reaction between them, the levels of the two IgM antibodies were actually correlated in serum (r = 0.6070; *P* = 0.0002. Fig 4A). The correlations between IgG and IgM anti-Kme3 were much smaller (r = 0.3334; *P* = 0.0469. Fig 4B).

We also found that levels of anti-Kme antibodies were strongly correlated with total serum IgM (r = 0.7027; *P*< 0.0001. Fig 4C). Correlations between IgM anti-Kme3 antibodies and total IgM were lower but still significant (r = 0.4622; *P* = 0.0059, Fig 4D). There was no correlation between IgG anti-Kme3 and total serum IgG (*P* = 0.2620, Fig 4E).

### Anti-Kme and anti-Kme3 antibodies appear in infants and are maintained throughout life

**Fig 5:**
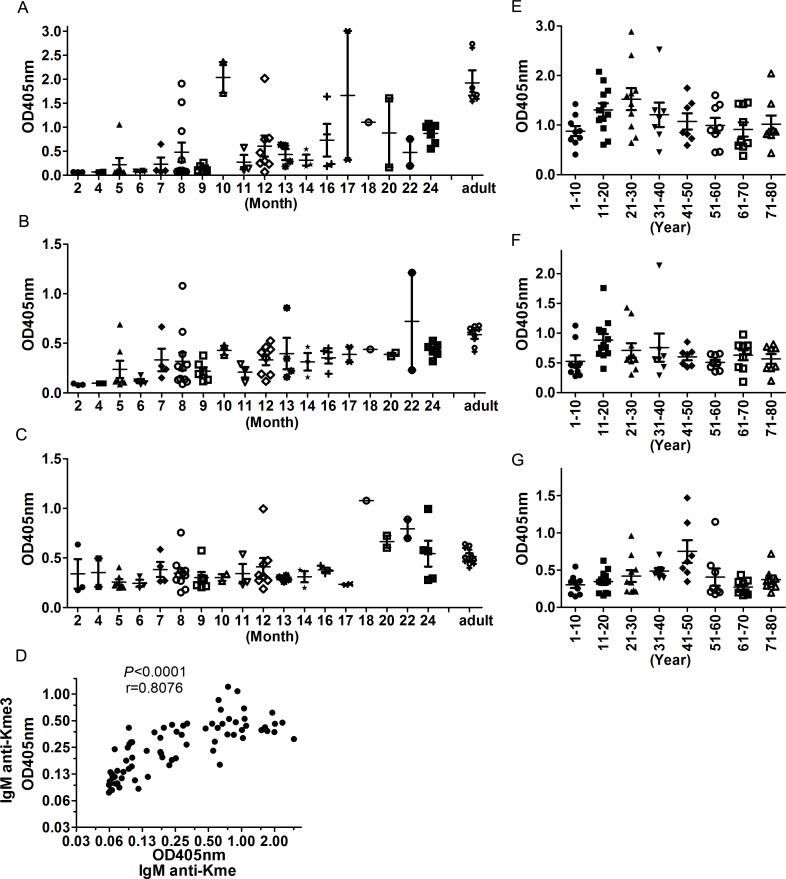
Changing of serumanti-Kme and anti-Kme3 antibody levels with age. Anti-Kme and anti-Kme3 antibody levels in sera from infants or donors of different ages were detected by ELISA using GGKme and GGKme3 conjugated BSA to coat plates. Serum samples were diluted 1:100. Anti-Kme and anti-Kme3 antibodies in serum were detected using KT47 anti-human IgG and KT16 anti-human IgM as primary antibodies and HRP conjugated goat anti-mouse IgG as secondary antibodies.(A) Serum levels of IgM anti-Kme in infants. (B) Serum levels of IgM anti-Kme3 in infants. (C) Serum levels of IgG anti-Kme3 in infants. (D) Correlations between IgM anti-Kme and IgM anti-Kme3 levels in infants.The correlations were analyzed by Spearman rank correlation analysis. (E) Serum levels of IgM anti-Kme in people of different ages. (F) Serum levels of IgM anti-Kme3 in people of different ages. (G) Serum levels of IgG anti-Kme3 in people of different ages.

To determine when anti-Kme and anti-Kme3 antibodies were first expressed, we tested serum samples of babies aged 1 month to 2 years old. We found that IgM anti-Kme antibodies could be detected in some babies aged 5 months (Fig 5A). All babies who reached age two could express the antibodies although their levels were lower compared with the IgM in adults. Anti-Kme3 IgM showed the same trend, i.e., the earliest appearance of the IgM was in babies aged 5 months, and all babies could produce the IgM when they reached age two (Fig 5B). In comparison, anti-Kme3 IgG could be detected much earlier (Fig 5C), which was likely caused by the transfer of IgG from mothers to their babies during pregnancy. Similarly to those in adults, serum levels of IgM anti-Kme and Kme3 also correlated well in infants (r = 0.8076; *P*< 0.0001, Fig 5D), which implied that the two antibodies appeared simultaneously.

We also measured levels of anti-Kme and anti-Kme3 antibodies in people aged one year to eighty years and found that both IgM anti-Kme and anti-Kme3 antibodies were maintained throughout life and reached high levels after adolescence and slightly declined after 40 years old (Fig 5E and 5F). The pattern of IgG anti-Kme3 was different. Peak levels were reached between ages 40 and 50 (Fig 5G).

### Both IgM anti-Kme and Kme3 antibodies are reduced in SLE patients

**Fig 6:**
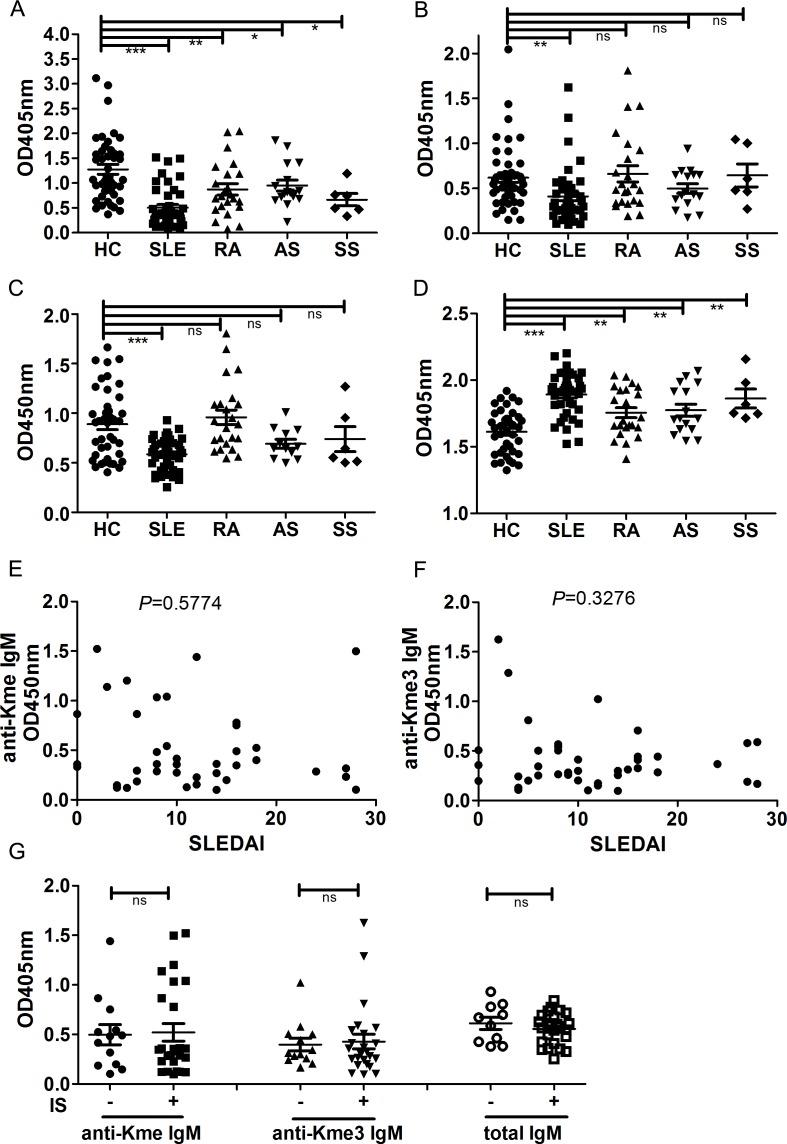
Comparison of IgM anti-Kme and anti-Kme3 antibodieslevels in healthy subjects and rheumatic diseasespatients. Ninety-six-well plates were coated with GGKme and GGKme3 conjugated BSA. Serum samples were 1:100 diluted and tested. Healthy subjects (n = 44), SLE patients (n = 42), RA patients (n = 23), AS patients (n = 16) and SS patients (n = 6). KT47 anti-human IgG and KT16 anti-human IgM were used as primary antibodies, and HRP conjugated goat anti-mouse IgG was used as a secondary antibody. *P* values were calculated using Student’s *t* test. *P< 0.05, **P< 0.01, ***P< 0.001, ns, not significant. The correlations were analyzed by Pearson correlation analysis. (A) Levels of IgM anti-Kme antibodies. (B) Levels of IgM anti-Kme3 antibodies. (C) Levels of total IgM antibodies. (D) Levels of total IgG antibodies. (E) Correlations between SLEDAI scores and levels of anti-Kme IgM. (F) Correlations between SLEDAI scores and levels of anti-Kme3 IgM. (G) Levels of anti-Kme IgM and anti-Kme3 IgM in immunosuppressant treated and untreated SLE patients.

We previously reported that serum levels of IgM anti-Kme were significantly lower in SLE patients than healthy subjects [12]. To determine whether IgM anti-Kme levels were also low in other rheumatic diseases, we tested sera from patients with RA, SS and AS. Our results showed that patients with other rheumatic diseases also had lower levels of IgM anti-Kme, but the reduction was not as significant as those observed in SLE patients (Fig 6A). The levels of IgM anti-Kme3 in SLE were also significantly lower than those in healthy subjects, whereas the antibody levels in patients with other rheumatic diseases were not significantly low (Fig 6B). The levels of total IgM in SLE patients were also lower than those in healthy subjects (Fig 6C), whereas the levels of total IgG in SLE and other rheumatic patients were higher than those in healthy subjects (Fig 6D). As previously reported [12], there was no correlation between the levels of anti-Kme IgM and disease activity of SLE (*P* = 0.5774, Fig 6E). There was also no correlation between the levels of anti-Kme3 IgM and disease activity of SLE (*P* = 0.3276, Fig 6F). In addition, treatment of SLE patients with immunosuppressants such as cyclophosphamide, cyclosporin A and methotrexate seemed to have no effect on the levels of anti-Kme IgM, anti-Kme3 IgM and total IgM (Fig 6G).

### Anti-Kme and anti-Kme3 antibodies can bind NETs

**Fig 7:**
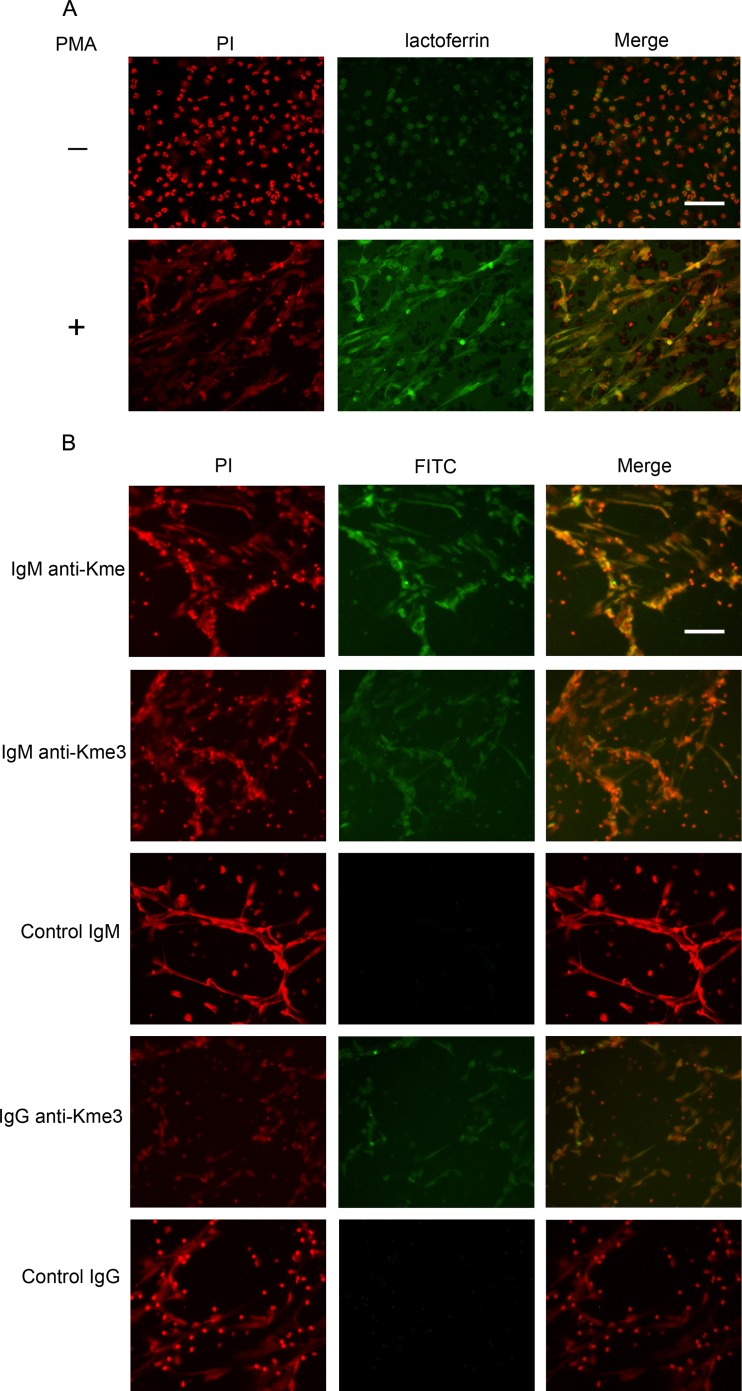
Binding of anti-Kme and anti-Kme3 antibodies to NETs. Neutrophils were isolated from healthy donors and stimulated with PMA for NETs formation (see Materials and Methods). DNA was stained using PI (red color), and lactoferrin was stained using FITC conjugated anti-lactoferrin antibody. After PMA treatment, neutrophils were incubated with affinity purified anti-Kme and anti-Kme3 antibodies. KT16 (anti-IgM) and KT47 (anti-IgG) were used as primary antibodies, and FITC conjugated goat anti-mouse IgG was used as a secondary antibody. IgM and IgG anti-NP antibodies were used as isotype controls. The result shown is a representative of three separate experiments. Scale bars, 50 μM. (A) PMA induced neutrophil release. (B) Binding of anti-Kme and anti-Kme3 antibodies to NETs. The results shown are representative of three separate experiments.

Neutrophil extracellular traps (NETs) are materials containing chromatin and granular proteins and are released from neutrophils during cell activation [15]. The lysines of histone in NETs are methylated, such as K9, K27, K36, etc [16]. Therefore, we test the interaction between NETs and anti-Kme or anti-Kme3 antibodies. Using FITC conjugated anti-lactoferrin antibody to visualize granular proteins and PI to visualize DNA, the results showed that there was no NETs release when neutrophils were not activated, and a large amount of NETs were released when neutrophils were treated with PMA (Fig 7A).

We further analyzed binding of anti-Kme and anti-Kme3 antibodies to NETs. The results showed that both antibodies could efficiently bind to NETs, whereas control antibodies could not (Fig 7B).

## Discussion

In current and previous work, we identified two antibodies against very small epitopes with only one amino acid. One was against mono-methylated lysine mostly of the IgM isotype. The other was against tri-methylated lysine of both IgM and IgG isotypes. Although their epitopes were small and similar, the antibodies did not cross-react with each other. Thus, they were different antibodies. However, these two antibodies shared many similarities. First, neither was produced at birth, and their earliest appearance was around 5 months after birth. Second, both were expressed in all healthy human subjects older than two years old and maintained thereafter throughout life. Third and most importantly, their levels were closely correlated in serum. All these findings indicated that the antibodies were produced by cells from a same B cell subset.

IgM is produced by three subsets of B cells, i.e., B1 B cells, follicular B cells (FO B cells) and marginal zone B cells (MZ B cells) [17]. B1 B cells are generated during early ontogeny and are associated with innate immunity. These cells are the major source of IgM NAAs [18]. FO B cells, which belong to the family of B2 B cells, are responsible for adaptive immunity and produce IgM antibodies when they first encounter foreign antigens. They then switch to produce IgG or other antibody isotypes during secondary or subsequent antigen challenges [19]. MZ B cells, which also belong to the family of B2 B cells, not only produce antibodies after infection but also produce NAAs under homeostatic conditions [20].

The IgM anti-Kme and anti-Kme3 antibodies are not likely produced by B1 or B1-like B cells in humans because they are not produced in newborn babies. The antibodies are not likely produced by FO B cells either because FO B cells usually produce antibodies against non-self antigens and their antibody levels fluctuate in the presence or absence of antigen stimulation [21]. We believe that the anti-Kme and anti-Kme3 antibodies are likely produced by MZ B cells. It is known that MZ tissue is not fully formed until 1–2 years old in humans, and MZ B cells were observed in the spleen of an 8-month old child [22]. Thus, antibodies produced by MZ B cells can not appear in early months after birth. To prove the antibodies were produced by MZ cells, we performed ELISPOT assays. Because human spleens were difficult to obtain and MZ B cells could go into circulation [23], we isolated PBMCs from human peripheral blood and tested their reaction to GGK-, GGKme- and GGKme3-coated membranes. We found that GGK-peptide-coated membranes never had positive spots, whereas GGKme- and GGKme3-coated membranes always had IgM but not IgG positive spots (S1 Fig). However the spots were too few to make a conclusion.

We found that a large proportion of anti-Kme3 antibodies had switched to IgG and, in contrast, anti-Kme antibodies hardly had any class switching from IgM to other isotypes. Class switching is regulated by T cells and is affected by environmental conditions, especially by cytokines [24]. This switching typically occurs in germinal centers for FO B cells. Nonetheless, MZ B cells can also undergo class switching. MZ B cells participate in both T cell independent and dependent immune responses and can continuously shuttle between marginal zone and lymphoid follicles [25],which provides them opportunities to contact T cells and undergo class switching under the effects of T cells. We do not know why anti-Kme and anti-Kme3 B cells selectively switch. We speculate that lysine mono-methylation is associated with biological processes through which little cell debris is generated and can be easily cleaned through complement activation after IgM antibodies bind to their targets, whereas lysine tri-methylation is associated with biological processes through which a large amount of cell debris is generated [26], which needs a more robust way to remove cell debris. Binding IgG to its target cannot only activate complement but can also stimulate phagocytes to engulf cell debris.

Both Kme and Kme3 are very small epitopes. We guessed that antibodies recognizing such a small epitope should have similar or even the same variable regions. To determine whether this reasoning was true, we performed an MS analysis using affinity purified IgG and IgM antibodies from three randomly selected donors. The results confirmed our speculation. For anti-Kme3 antibodies, all three individual donors had variable regions that were derived from the IGHV3-7 gene segment. Because the usage rate of IGHV3-7 was only 4% in the normal human B cell repertoire [27], the probability of having this gene segment appear in all three affinity purified samples was little. Thus, the variable regions of anti-Kme3 antibodies from the three subjects did share similar structures. For anti-Kme antibodies, all three samples also had shared variable regions derived from the IGHV3-7 gene segment. The MS also identified more sequences from the IGHV3-74 and IGHV2-5 segments. The usage rates of IGHV3-74 and IGHV2-5 are only 2% and 3%, respectively, in the normal B cell repertoire [27,28]. Thus, the anti-Kme antibodies should also have limited variable regions.

The MS analysis showed that both IgM anti-Kme and IgG and IgM anti-Kme3 antibodies in three people had conserved CDR2 as the germ line sequences of IGHV3-7/74. Furthermore, *de novo* sequencing revealed that their CDR1 sequences were similar, although they were not completely conserved as the germ line sequences. A distinctive mutation occurred at the double serine positions, which resulted in the replacement of one of the serine residues by a basic amino acid, i.e., arginine or lysine. Thus, we can conclude that both anti-Kme and anti-Kme3 antibodies have essentially the same CDR1 and CDR2 and the only differences must have been located in the CDR3 region to allow the two antibodies to distinguish between Kme and Kme3 without cross-reaction.

The anti-Kme and Kme3 antibodies appear to have a combination of the properties of NAAs and immune autoantibodies. Similarly to NAAs, the antibodies exist in all healthy subjects and their levels are correlated with total IgM, indicating that the antibodies are constantly and steadily expressed under homeostatic conditions. However, they do not show the typical properties of NAAs. They are not produced in neonates. NAAs are typically of the IgM isotype, whereas the majority of anti-Kme3 antibodies are IgG. Anti-Kme and anti-Kme3 antibodies are not polyreactive but monoreactive with precise antigen specificities. Nevertheless, polyreactivity is not a prerequisite for NAAs, and NAAs may also be monoreactive [29]. In addition, Kme and Kme3 can appear on different histones or other molecules [30]. Antibodies against Kme and Kme3 would appear polyreactive if they are recognizing their epitopes on different proteins.

The anti-Kme and anti-Kme3 antibodies exist in all healthy subjects at reasonable concentrations (approximately 5 μg anti-Kme and approximately 7 μg anti-Kme3 per milliliter serum on average). An obvious question is what their functions are. Although lysine methylation occurs inside of cells, anti-Kme and anti-Kme3 antibodies must bind their targets outside of cells either on the surface of apoptotic cells or when released into the surrounding environment. One type of structure that behaves in this manner is NETs [16], which are substances released from neutrophils during cell activation [15]. We found that both anti-Kme and anti-Kme3 antibodies could bind NETs. Therefore, they can act as opsonins in the clearance of NETs or other materials that contain mono- or tri-methylated lysine and released into body fluid during biological processes. It has been reported that SLE patients are prone to produce NETs but have defects with respect to the clearance of NETs, which in turn are autoantigens in this disease [31,32]. Low levels of anti-Kme and anti-Kme3 antibodies in SLE and other rheumatic diseases are a disadvantage in the clearance of NETs.

We found that the levels of IgM anti-Kme and anti-Kme3 antibodies in SLE patients were significantly lower than those in healthy control subjects. The cause of the low levels is not clear. It has been reported that SLE patients tend to have low total IgM [33] and have defects on MZ cells [34], which may affect the production of IgM anti-Kme and anti-Kme3 antibodies. We also found that the low antibody levels in SLE patients did not correlate with the SLEDAI scores [12], which is not surprising because SLE is a disease that is affected by many factors.

In conclusion, anti-Kme and anti-Kme3 antibodies have distinct but closely related properties. The antibodies are likely generated from the same B cell subset and exhibit similar functions in the body.

## Supporting information

S1 Fig Detection of anti-Kme and anti-Kme3 secreting B cellsin blood by ELISPOT. MultiScreen IP filter plates (96-well) were coated with 1 μg/well GGK-BSA, GGKme-BSA or GGKme3-BSA in PBS at 4°C overnight. After washed with PBS, the plates were blocked with 200 μL/well of RPMI 1640 medium containing 10% fetal bovine serum (FBS). PBMCs from healthy volunteers were isolated from heparinized blood by Ficoll lymphocyte separation medium and suspended in RPMI 1640 medium containing 10% FBS, 10 μg/mL LPS, 50 ng/mL PMA. The isolated PBMCs (10^6^) were added to each well and cultured for 24 h. Then, the cells were removed, and the plates were washed. KT47 anti-human IgG and KT16 anti-human IgM were used as primary antibodies, and HRP conjugated goat anti-mouse IgG was used as a secondary antibody. Color development was performed using AEC reagent (DAKEWE Biotech, Beijing, China) as the substrate. The reaction was stopped by washing with distilled water, and the plates were left to dry until they were counted. The spots were read by CTL ImmunoSpot S5 analyzers (Cellular Technology, Shaker Heights, OH, USA).The result shown is a representative of three separate experiments.
(TIF)
