# Supplementary figures and images for: Autoantibodies against mono- and tri-methylated lysine display similar but also distinctive characteristics
Source: PLoS One. 2017 Feb 21;12(2):e0172166. doi: 10.1371/journal.pone.0172166 (PMC5319698; doi:10.1371/journal.pone.0172166)

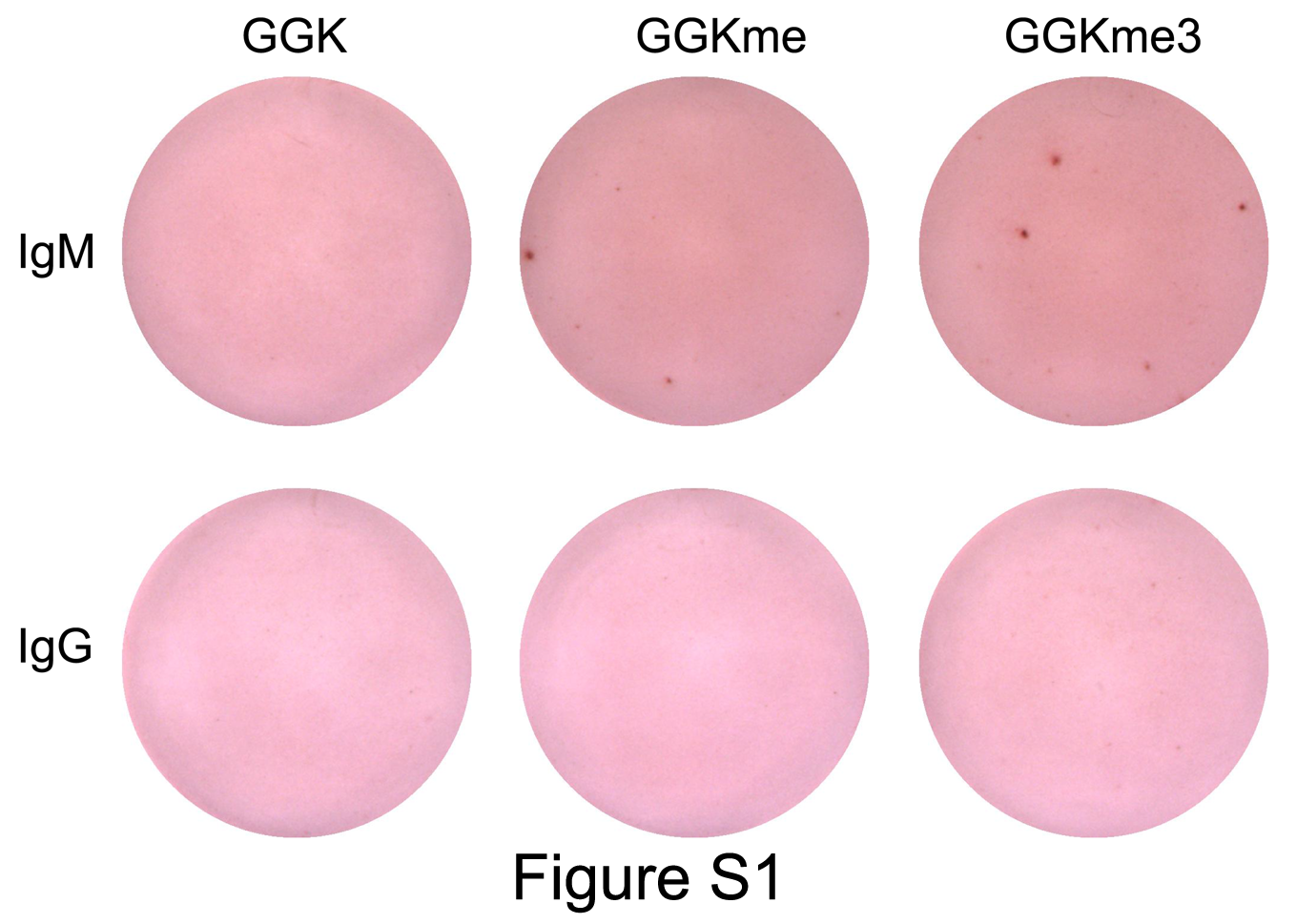

Supplement: S1 Fig — MultiScreen IP filter plates (96-well) were coated with 1 μg/well GGK-BSA, GGKme-BSA or GGKme3-BSA in PBS at 4°C overnight. After washed with PBS, the plates were blocked with 200 μL/well of RPMI 1640 medium containing 10% fetal bovine serum (FBS). PBMCs from healthy volunteers were isolated from heparinized blood by Ficoll lymphocyte separation medium and suspended in RPMI 1640 medium containing 10% FBS, 10 μg/mL LPS, 50 ng/mL PMA. The isolated PBMCs (106) were added to each well and cultured for 24 h. Then, the cells were removed, and the plates were washed. KT47 anti-human IgG and KT16 anti-human IgM were used as primary antibodies, and HRP conjugated goat anti-mouse IgG was used as a secondary antibody. Color development was performed using AEC reagent (DAKEWE Biotech, Beijing, China) as the substrate. The reaction was stopped by washing with distilled water, and the plates were left to dry until they were counted. The spots were read by CTL ImmunoSpot S5 analyzers (Cellular Technology, Shaker Heights, OH, USA).The result shown is a representative of three separate experiments. (TIF) [file pone.0172166.s001.tif]
